# Energy Absorption Mechanisms in Minimal Surface Versus Truss-like Lattice Structures: Experimental and Numerical Insights

**DOI:** 10.3390/ma19091823

**Published:** 2026-04-29

**Authors:** Zhou Huang, Yong Liu, Junwei Liu, Dong Mu

**Affiliations:** Institute of Systems Engineering, China Academy of Engineering Physics, Mianyang 621900, China; huangzcaep@163.com (Z.H.); liuyonghust@126.com (Y.L.);

**Keywords:** specific absorption energy, additive manufacturing, minimal surface, lattice

## Abstract

Truss-like and minimal surface-based cells are among the promising candidates for novel impact-resistant structural designs. However, the influence of cell configurations on impact resistance performance remains unclear. In this paper, the energy absorption characteristics of three truss-like cells (BCC, Fluorite, and Diamond) and three minimal surface cells (Gyroid, Primitive, Diamond) are systematically compared using quasi-static compression experiments and refined numerical models. Experimental results indicate that minimal surface cells possess clearly superior specific energy absorption performance. Specifically, the Gyroid (G-surface) exhibits a specific energy absorption (25 kJ/kg) approximately 2.3 times greater than the highest value among truss-like cells (11 kJ/kg), accompanied by an extended plateau strain by about 20%. Additionally, due to stress concentration at joints, truss-like cells show notably lower plateau forces compared to minimal surface cells. However, truss-like cells demonstrate better manufacturing precision and quality control, as evidenced by a relatively small average weight deviation (about 1.2%). Furthermore, numerical simulations were conducted to explore differences in deformation mechanisms between two representative cells. Results reveal that the BCC structure absorbs energy through localized shear band formation induced by point plastic hinges, whereas the Primitive (P-surface) minimal surface structure achieves more uniform plastic deformation via distributed line plastic hinges. Finally, impact simulations of protective structures show that the maximum stress in the P-surface-filled structure is reduced by 4.6% compared to the BCC-filled structure, and stress distribution uniformity is improved by 37%. The findings from this study provide valuable references and data support for future anti-impact structural designs.

## 1. Introduction

Lattice structures have received extensive attention from impact protection designers due to their high specific strength, high specific stiffness, and excellent energy absorption capabilities [1,2,3]. Structures composed of periodically arranged lattice unit cells have been gradually replacing traditional metallic foams in various fields [4,5,6,7,8], such as aerospace buffer devices and vehicle collision energy absorption boxes, promoting the development of lightweight design towards multifunctional integration [9,10,11,12,13,14]. However, despite extensive research, a direct and fair comparison of their energy absorption performance under identical conditions remains scarce, hindering optimal selection for specific impact-resistant applications.

**Figure 1:**
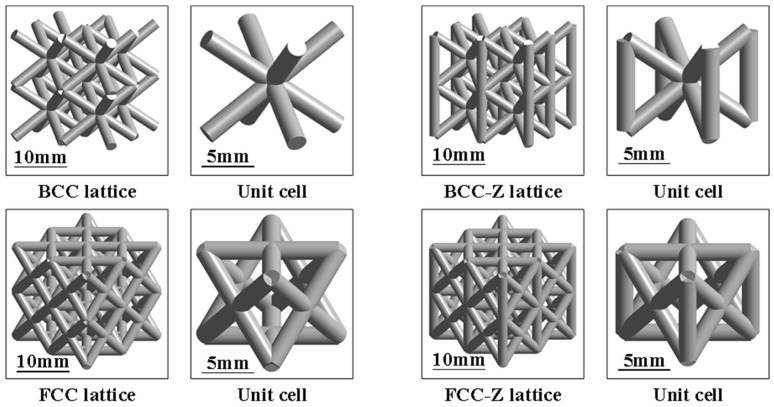
Typical truss-like lattices and cells.

Existing studies have established distinct deformation mechanisms for these two types of lattices. Truss-like unit cells achieve good force transmission through the axial loading of discrete rods [15]. Typical configurations include Body-Centered Cubic (BCC), Face-Centered Cubic (FCC), and their variants (BCC-Z/FCC-Z) [16,17,18], as shown in Figure 1. The degree of freedom at the nodes of truss-like unit cells leads to differences in deformation modes [19,20]: tension-dominated unit cells (such as Diamond) dissipate energy through axial yielding of rods, while bending-dominated unit cells (such as BCC) rely on plastic hinge rotation to achieve progressive collapse. Generally, unit cell configurations with bending deformation modes are more effective because their deformation process is relatively stable, with less tendency for local instability and oscillation. Zhang et al. [11] explored the influence of additional supports on the mechanical properties and energy absorption of self-supporting structures with triple, quadruple, and sixfold rotational symmetry by adding boundary vertical struts and in-plane diagonal struts, using theoretical models, experimental characterization, and finite element methods for systematic research. Li et al. [21] changed the degree of freedom of lattice structure nodes by adding additional supports, transforming the bending-dominated deformation mode into a tension-dominated one to obtain more excellent mechanical properties and energy absorption effects. Bogusz et al. [22] designed five lattice structures with different topological cross-sections using durable resin as a 3D printing material, conducted quasi-static axial compression experiments, analyzed their failure modes, and compared the performance of lattice structures, such as initial peak force, peak force efficiency, average platform force, and specific energy absorption.

**Figure 2:**
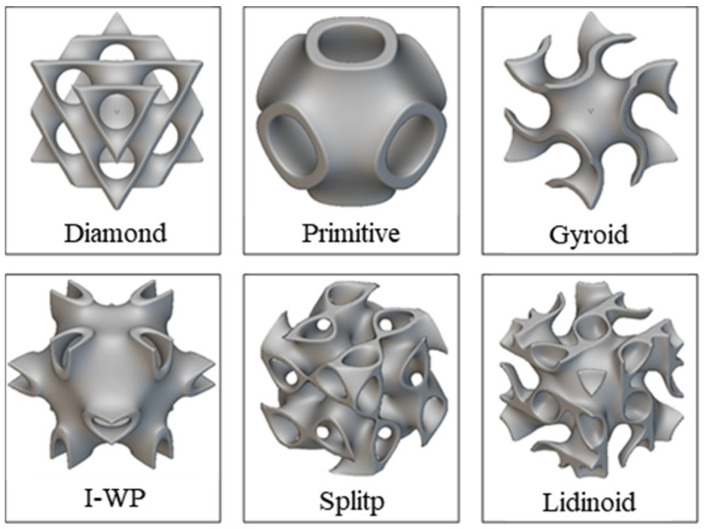
TPMS configuration.

Triply Periodic Minimal Surfaces (TPMS) (as shown in Figure 2) are constructed based on continuous surfaces with zero mean curvature, and their implicit equations endow the unit cell configurations with natural structural continuity and stress uniformity [23,24,25,26]. This mechanical feature enables the load within the unit cell to be rapidly dispersed to a larger area, thus effectively overcoming the stress concentration problem prone to occur in traditional truss-like unit cells. Meanwhile, TPMS unit cells can also conveniently control the unit cell type and size by adjusting the parameters of the implicit modeling function. Due to the above advantages, minimal surface unit cells have been widely studied. Zhao et al. [27] proposed a novel surface offset design method aimed at improving the mechanical strength and energy absorption performance of TPMS lattice structures. In the study, four typical TPMS lattice structures, including Diamond, Initial Lattice, Spiral Icositetrahedron, and Body-Centered Cubic, were designed, and their mechanical behaviors were evaluated using the Johnson-Cook simulation analysis model. Guo et al. [28] redefined the shape parameters based on the classical Primitive surface, took the opening diameter as one of the variables, and investigated the compressive strength and specific energy absorption of P-surface lattice structures with different opening directions under quasi-static compression conditions. Furthermore, Sun et al. [29] used titanium alloy as the material to fabricate panel-free lattice structures of three surfaces, namely Primitive, Gyroid, and Diamond, and conducted defect analysis on the experimental components through SEM. Shinde et al. [30] took aluminum alloy as the material to analyze the specific energy absorption and fluctuation of compressive reaction force of 2D honeycomb structures, two 3D truss-like lattice structures, and three 3D minimal surface lattice structures.

The application of lattice unit cells in energy-absorbing structures faces a key challenge: while numerous studies focus on innovative configurations, a multidimensional comparison of performance across different unit cell types remains scarce. Although energy absorption data for various designs can be found in the literature, inconsistent reporting conditions—such as differing processing parameters, materials, and heat treatments—make a fair comparison difficult. This lack of standardized benchmarking hinders the effective selection of optimal unit cell configurations for specific applications.

To address this issue, this paper systematically compares the energy absorption characteristics of typical truss-like unit cells and minimal surface unit cells based on quasi-static compression tests. Three typical configurations are selected for each type, and they are processed and manufactured using the same batch of 316L stainless steel material to ensure consistent processing parameters. Each unit cell undergoes two repeated experiments to ensure the validity and comparability of experimental data. Subsequently, a simulation analysis model corrected by experimental data is established. Based on the simulation results, the differences in deformation modes of different types of unit cells are analyzed and compared, and the causes of differences in energy absorption performance are revealed. Finally, the performances of impact-resistant structures based on the two types of unit cells are compared through simulation analysis of impact-resistant structures. The relevant research results can provide design references and data support for the subsequent design of impact-resistant structures.

## 2. Comparison of Truss-Type and Minimal Surface Unit Cell Configurations

**Figure 3:**
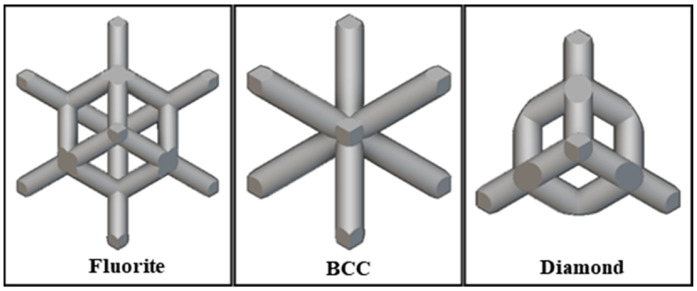
Three types of single-cell structure diagrams.

This study selects three typical truss unit cells: Body-Centered Cubic (BCC), Fluorite, and Diamond configurations (Figure 3). Based on differences in deformation mechanisms during compression, truss-type unit cells can be divided into two categories:

(a) Bending-dominated type: The rods are primarily subjected to bending deformation. According to Ashby’s formula, the equivalent Young’s modulus (E*) and relative density (ρ) satisfy a power-law relationship, i.e., $E*\propto \rho^{n}$ (*n* ≈ 1.5−2.0).
(b) Tension-dominated type: The rods mainly bear axial tensile and compressive loads, and the mechanical properties show a linear relationship $E*\propto \rho^{n}$.

The dominant deformation mode of unit cells can be predicted by Maxwell’s static criterion [16]:

(1) M=S−3n+6,

In the formula, *S* is the total number of rods in the unit cell, and *n* is the number of nodes. If *M* < 0, the structure is bending-dominated; if *M* = 0, it is tension-dominated.

On the other hand, for minimal surface unit cells, this paper focuses on three types of configurations: P-surface (Primitive), G-surface(Gyroid), and D-surface(Diamond). The geometric characterization method of minimal surfaces is relatively complex, and there are currently two common methods:

(a) Enneper-Weierstrass parameterization method: The geometric configuration of the minimal surface is accurately calculated through the explicit function of Equation (2) [8],

(2) x=Reeiθ∫ω0ω1−τ2Rτdτy=Reeiθ∫ω0ωi1+τ2Rτdτz=Reeiθ∫ω0ω2τRτdτ,

where i^2^ = −1, τ is a complex variable, θ is the Bonnet angle, and Re denotes the real part of the complex variable. R(τ) is the Weierstrass function for different types of TPMS units. The Weierstrass functions for D-surface, P-surface, and G-surface can be expressed as

(3) $$R\left(\tau \right)=\frac{1}{\sqrt{\tau^{8}-14\tau^{4}+1}},$$

The Bonnet angles of D-surface, P-surface, and G-surface are 0°, 90°, and 38.0147°, respectively [8]. However, this method can generally only generate a few types of TPMS unit cells.

(b) Iso-surface method: The patch geometric model is obtained by calculating the iso-surface of a given function,

(4) $$\phi \left(r\right)=\sum_{k=1}^{K}A_{k}\cos\left[\frac{2\pi \left(h_{k}\cdot r\right)}{\lambda_{k}}+P_{k}\right]=C,$$

where $A_{k}$ is the amplitude, $\lambda_{k}$ is the periodicity factor, and $P_{k}$ is the function phase. For P-surface, D-surface, and G-surface unit cells, the corresponding implicit functions are

(5) cos(x)+cos(y)+cos(z)=t,

(6) sin(x)·sin(y)·sin(z)+sin(x)·cos(y)·cos(z)+cos(x)·sin(y)·cos(z)+cos(x)·cos(y)·sin(z)=t,

(7) cos(x)·sin(y)+cos(y)·sin(z)+cos(z)·sin(x)=t.

## 3. Experiments and Results

### 3.1. Specimen

The energy absorption capacity of lattice structures primarily depends on their deformation modes (such as plastic buckling and strut yielding) and the macroscopic stress–strain response. The plateau stress and densification strain observed in quasi-static compression tests serve as key indicators for evaluating their impact resistance [31]. Studies indicate that while strain rate effects may lead to increased dynamic strength, the energy absorption efficiency (e.g., specific energy absorption) and deformation mechanisms (such as uniform deformation versus localized shear bands) revealed by quasi-static experiments are highly correlated with performance under dynamic impact. For instance, the dynamic plateau stress of auxetic lattices and graded TPMS structures can be effectively predicted using quasi-static data [32]. Therefore, quasi-static compression experiments provide a reliable basis for rapidly assessing the impact resistance potential of lattice structures.

**Figure 4:**
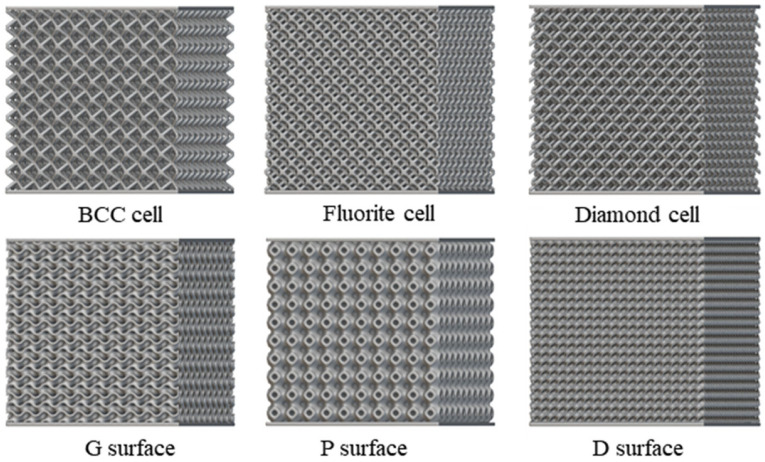
Experimental test objects, including BCC, Fluorite, Diamond, G-surface, P-surface, D-surface.

In this study, 316L stainless steel powder (particle size 15–53 μm) was used to fabricate lattice compression specimens via Selective Laser Melting (SLM) technology. The unit cell size was uniformly set to 5 mm × 5 mm × 5 mm (length × width × height). To eliminate the influence of boundary effects on mechanical responses, the test specimens were designed as 10 × 10 × 10 periodic arrangements of unit cells (i.e., 10 unit cells repeated along the X/Y/Z directions), with solid end plates of 1 mm thickness added to the top and bottom of the array for uniform transmission of compressive loads (Figure 4). Thus, the overall dimensions of the experimental specimen were 50 mm (length) × 50 mm (width) × 52 mm (height), where the height direction included the thickness of the end plates (2 × 1 mm). Detailed processing parameters are listed in Table 1.

**Figure 5:**
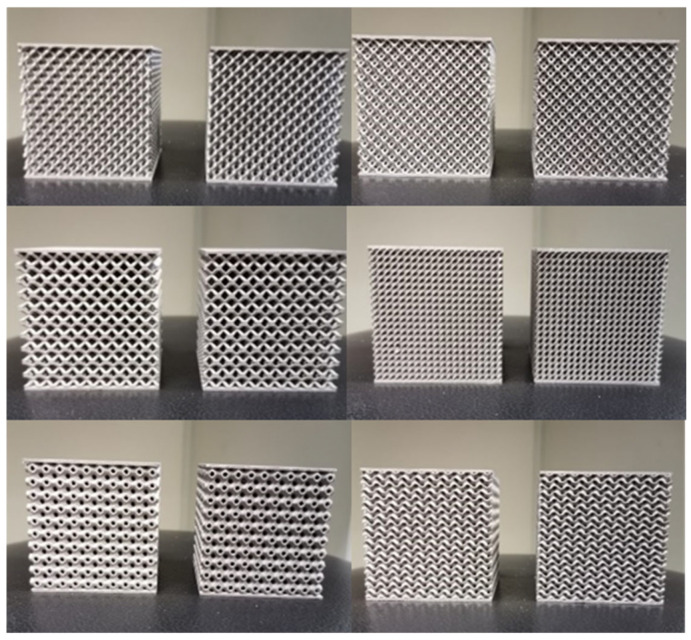
Photographs of Six Types of Lattice Test Specimens.

The processed experimental specimens are shown in Figure 5. Considering the characteristics of additive manufacturing, a 0.5 mm allowance was reserved on the bottom surface of the specimens, which was removed to ensure consistent processing quality.

**Figure 6:**
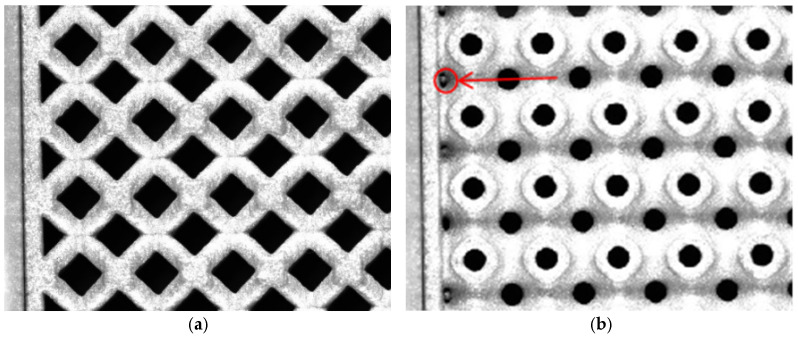
Local enlarged view of test specimens: (**a**) BCC type; (**b**) P-surface type.

As shown in Table 2, although the theoretical weight of the STL models for all six unit cell specimens was 287.6 g, their measured weights exhibited significant configuration-dependent deviations: the average weight deviation of truss-like unit cells was only 6.9%, while that of TPMS-type unit cells reached 22.0%. The main reason for this weight deviation is the difference in the accessibility of powder removal channels. In truss-like unit cells, the discrete rods form an open structure, allowing the unmelted powder to be removed by high-pressure air gun cleaning. In contrast, the continuous surfaces of TPMS-type unit cells result in semi-enclosed cavities, where the unmelted powder remains after cleaning (Figure 6a). This practical challenge necessitates a thorough evaluation when applying TPMS structures in weight-critical applications like aerospace. Potential strategies to mitigate this issue include the design of dedicated powder-removal channels during the digital modeling stage and the adoption of more aggressive post-processing techniques. The final design decision must carefully weigh the theoretical mechanical superiority of TPMS against the achievable manufacturing precision and weight control.

Figure 6 compares the powder removal effects of BCC and Primitive surface specimens. Sintered powder nodules were observed at the junction of the top plate and unit cells in the Primitive specimen, whereas no visible residue was found in the nodal regions of the BCC specimen. Therefore, truss-like unit cells are a more reliable choice for applications requiring strict weight control.

### 3.2. Experimental Methods

**Figure 7:**
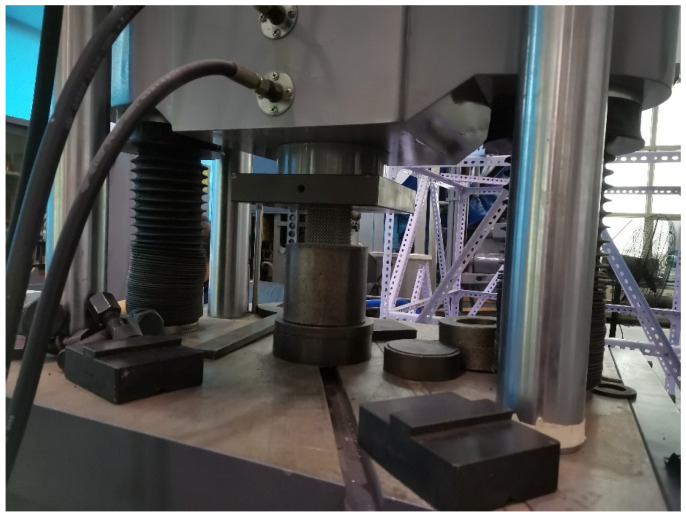
Electronic universal testing machine.

The compression test was conducted in accordance with standards using a microcomputer-controlled electronic universal testing machine, as shown in Figure 7. Each lattice specimen was placed horizontally at the center of the compression fixture for testing. The specimen was compressed axially at a constant rate of 5 mm/min, with a maximum compression displacement set to 35 mm, corresponding to an engineering strain ε = Δ*L*/*L*_0_ = 35/52 ≈ 67.3%. The testing machine was equipped with a bidirectional hinged universal joint to eliminate initial centering errors, ensuring the axial load eccentricity <1%. A built-in force sensor recorded the force data, and the displacement (specimen shortening) of the fixture was recorded at a frequency of 1 Hz. The universal joint in the loading section of the testing machine was used to reduce the influence of load eccentricity. Raw data were processed by a five-point cubic smoothing filter to calculate the engineering stress–strain curve (*σ* = *F*/*A*_0_, *ε* = Δ*L*/*L*_0_, where *A*_0_ = 50 × 50 mm^2^ is the initial cross-sectional area of the specimen).

### 3.3. Experimental Results

**Figure 8:**
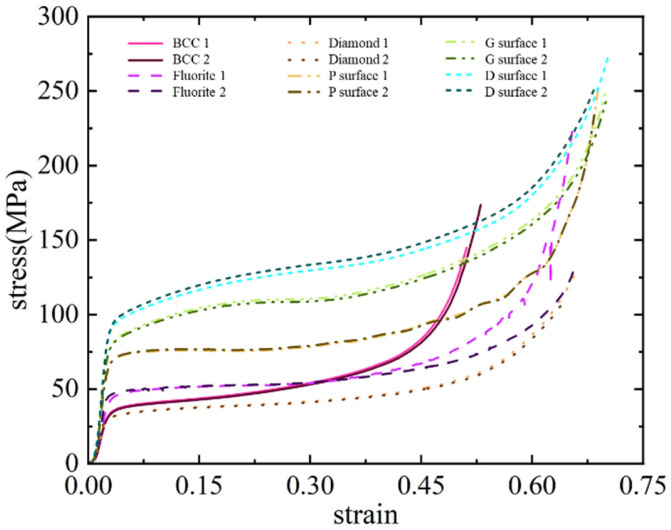
Compression curve of 6 different types of cells.

Figure 8 shows the compression curves of experimental specimens with six different unit cell configurations, each tested twice with good repeatability of experimental data. Results show that the compression curves of different types of lattice unit cell specimens all exhibit typical three-stage characteristics: linear elastic stage (strain *ε* < 5%), plateau stage (5% < *ε* < 60%), and densification stage (*ε* > 60%). It can be found that configuration differences lead to significant differentiation of plateau forces: the plateau stress of truss-like unit cells (approximately 50 MPa) is significantly lower than that of minimal surface unit cells (75–125 MPa). The main reason for this phenomenon is that truss-like unit cells are composed of beams connected at nodes, where stress concentration easily causes local high stress, forming point plastic hinges that trigger plastic flow. In contrast, minimal surface unit cells are composed of continuous surfaces with weak internal stress concentration, which require the formation of continuous line plastic hinges to induce overall plastic flow of the unit cell, thus requiring greater external loads and resulting in higher plateau stress.

**Figure 9:**
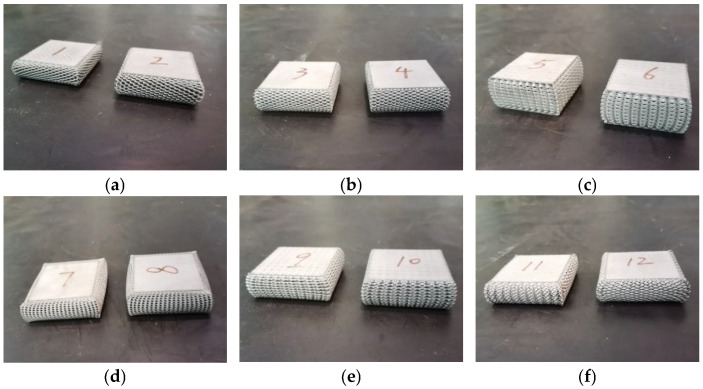
Compression state of the test: (**a**) fluorite monosomes; (**b**) diamond-type monocytes; (**c**) BCC monosomes; (**d**) G curved cell; (**e**) P curved cell; (**f**) D surface cell.

Figure 9 shows the compaction states of the six experimental specimens. Specimens with different unit cell types all exhibit a certain degree of deformation overflow, where the internal lattice structure extrudes outward in all directions after compression. This overflow deformation mode helps delay the onset of the densification stage. Among various unit cells, the BCC unit cell shows the least compression overflow deformation, implying that it enters the densification stage earlier during compression—this explains why the BCC unit cell’s compression curve in Figure 8 enters the third stage first. In minimal surface unit cells, the P-surface unit cell specimen demonstrates relatively weak expansion, while the other two surface unit cell specimens exhibit significant expansion. It should be noted that the numerical labels 1–12 in Figure 9 serve solely to track the experimental sequence and do not influence the results.

### 3.4. Comparison of Energy Absorption Performance

This section compares the energy absorption of the six unit cells, as listed in Table 3. By introducing indices such as specific plateau stress and specific energy absorption, performance evaluation deviations caused by weight differences are eliminated. The plateau force is calculated as the mean plateau stress (MPa), defined as the average value of the stress over the plateau region between the yield strain and the densification strain.

(8) $$\sigma_{\mathrm{pl}}=\frac{\int_{\varepsilon_{y}}^{\varepsilon_{d}}\sigma (\varepsilon )d\varepsilon }{\varepsilon_{d}-\varepsilon_{y}},$$

where $\varepsilon_{d}$ is the densification strain, and $\varepsilon_{y}$ is the yield strain.

The energy absorption capacity is calculated as

(9) $$EA=\int_{0}^{\varepsilon_{d}}\sigma \left(\varepsilon \right)d\varepsilon ,$$

where σ(ε) is the compressive stress. Based on this, the specific energy absorption is calculated as

(10) $$SEA=\frac{EA}{m}$$

where *EA* is the structural energy absorption capacity, and m is the weight of the experimental specimen.

The analysis results show that in terms of specific plateau stress, TPMS-type unit cells are significantly superior to truss-like ones, with the G-surface reaching 365.8 ± 2.8 MPa/kg, an increase of approximately 85% compared to the optimal truss-like (Fluorite 197.6 ± 1.8 MPa/kg); in terms of specific energy absorption, the average *SEA* of TPMS-type unit cells is 22.4 ± 4.3 kJ/kg, 2.3 times that of truss-like cells (9.6 ± 1.8 kJ/kg), and the G surface ranks first with 25.4 ± 0.7 kJ/kg; in terms of plateau strain, the average *ε*_plat_ of TPMS-type unit cells is 54%, which is 23% longer than that of truss-like cells (*ε*_plat_ = 44%), which is related to their topological characteristics: the higher surface area-to-volume ratio of TPMS (S/V = 1.8 mm^−1^ vs. 0.7 mm^−1^ for truss-like) delays the densification process of rod/wall contact; in terms of plateau force stability, truss-like unit cells are generally better than minimal surface unit cells: the plateau force fluctuations of truss-like unit cells are all low, among which the Diamond configuration has the best plateau force stability; among TPMS-type unit cells, the Primitive surface has relatively low volatility, but the Gyroid and Diamond surfaces cause plateau stress hardening due to the cooperative expansion of plastic zones.

For protective structures, the stability of the plateau stage is one of the important indicators for evaluating energy absorption structures. In the compression process of truss-like unit cells, the displacement is caused by the rotational deformation of point plastic hinges at the intersection of beams, which means that no large-area new plastic zones will appear after the formation of plastic hinges. The minimal surface unit cells have better integrity. After part of the area enters the plastic state, continuing to apply compressive deformation will cause the plateau force to rise due to the expansion of the plastic zone or the contact of different surfaces. It is worth noting that the P-surface unit cell is a special case among the minimal surface unit cells. The P-surface unit cell has a relatively stable plateau stage, but its plateau stress level is lower than that of other surface unit cells, which means that the formation of internal plastic hinges is less difficult, and the structure is conducive to deformation development.

It should be pointed out that for most impact-resistant structures, the initial peak force is also a key indicator, but in this study, in order to ensure that the six unit cell test pieces have the same weight and meet the processing accessibility, the porosity is low, and the quasi-static compression loading speed is slow, so there is no obvious initial peak in the compression curve. Therefore, the absence of a distinct initial peak force in our compression curves is directly attributable to the combined effect of the specimens’ low porosity and the quasi-static loading conditions employed in this study. In addition, due to the good processing quality of the stainless steel test pieces, no local damage occurred during the compression process. These factors together lead to the absence of an obvious oscillation stage in the compression curve, and thus no obvious initial peak force.

**Table 3:** Comparison of energy absorption performance of 6 different types of cells.

Lattice Type	Specific Plateau Stress/(MPa/kg)		SEA/(kJ/kg)		Plateau Strain	
	1	2	1	2	1	2
Fluorite	196.36	198.90	10.85	11.98	0.48	0.48
Diamond	162.35	162.33	9.43	9.62	0.46	0.47
BCC	159.08	157.30	7.37	7.34	0.37	0.37
G surface	362.95	368.61	24.89	25.87	0.55	0.57
P-surface	250.95	250.39	16.23	16.32	0.52	0.52
D surface	337.81	334.38	23.45	23.26	0.55	0.56

It is worth noting that the plateau force in the compression curve of the BCC unit cell shows an obvious strengthening phenomenon. This is because the overflow deformation of the BCC unit cell during the compression deformation process is small, resulting in its relatively earlier entry into the densification stage, which leads to a significant increase in the plateau stress. On the other hand, the Fluorite unit cell and the BCC unit cell are set to have the same volume fraction, and the former has more internal rods than the latter, so that the size of each rod in the BCC unit cell of the test piece is larger than that of the Fluorite unit cell, which also makes the rods in the BCC unit cell contact earlier, thus causing the increase in the plateau force.

**Figure 10:**
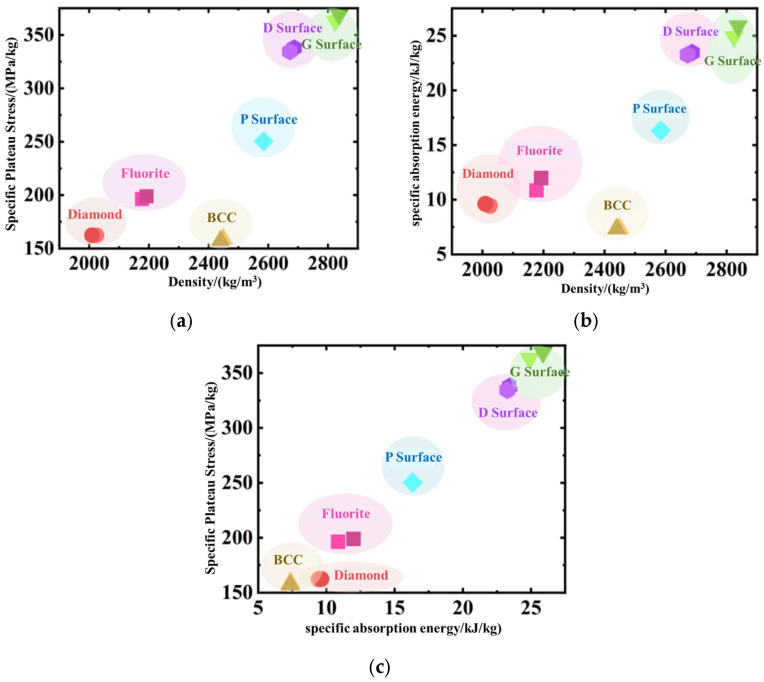
Comparison of the performance of different types of cells for impact energy absorption: (**a**) the relationship between specific platform force and equivalent density; (**b**) the relationship between specific absorption energy and equivalent density; (**c**) the relationship between specific platform force and specific energy absorption.

Figure 10 provides a comparative performance analysis of diverse unit cell configurations tailored for impact-energy-absorbing structural design requirements, incorporating metrics including specific energy absorption, specific plateau force, and relative density. These results reveal that the specific plateau force versus relative density plot (Figure 10a) demonstrates TPMS-based unit cells distributing predominantly in the upper region, signifying their superior stress transfer efficiency; the specific energy absorption versus relative density relationship (Figure 10b) illustrates the positive regulatory effect of continuous curved-surface topologies on energy absorption capabilities; while in the specific plateau force versus specific energy absorption plot (Figure 10c), a linear correlation exists between these two parameters across unit cells. It is worth noting that although certain energy-absorbing structural applications necessitate plateau force stability, other design scenarios impose less stringent stability requirements on plateau forces, thus mandating case-specific selection of infill unit cell architectures.

## 4. Numerical Simulation

Based on the comparative analysis above, the Body-Centered Cubic (BCC) and the P-surface topologies were selected as the representative unit cells for truss-based and sheet-based lattice structures, respectively. The BCC lattice serves as a classical, bending-dominated idealization of truss structures, while the P-surface, with its continuous morphology, exemplifies stretch-dominated behavior typical of sheet-based triply periodic minimal surfaces (TPMS). This selection facilitates a clear mechanistic comparison between the two fundamental lattice categories, minimizing redundancy as their deformation modes are distinctly different. The numerical models developed to simulate their mechanical response and energy absorption performance are detailed hereafter, starting with the BCC specimen.

### 4.1. Simulation Model of BCC Specimens

**Figure 11:**
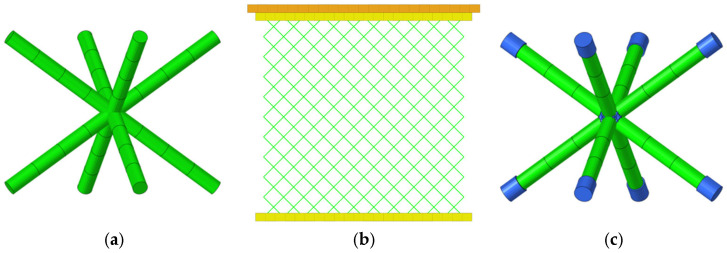
(**a**) BCC structural unit cell; (**b**) BCC-structured lattice; (**c**) BCC structural unit cell after bolding the intersection points of each beam.

In this section, a simulation model was developed for the body-centered cubic (BCC) configuration specimens, as shown in Figure 11a,b. The model features a unit cell size of 5 mm × 5 mm × 5 mm, beam diameter of 1 mm, and stainless steel plates with a thickness of 2 mm attached to the top and bottom surfaces. A bilinear isotropic hardening model was adopted for the material, with specific parameters listed in Table 1 (density 7850 kg/m^3^, Poisson’s ratio 0.31, elastic modulus 200 GPa, yield strength 480 MPa, tangent modulus 2 GPa). The BCC lattice was simulated using the Belytschko-Schwer beam element formulation. This explicit algorithm can produce a linearly varying moment along its length, offering more accurate elastic stress and enabling the detection of yielding at its ends. A mesh density of eight beam elements per strut was used [33], as illustrated in Figure 11.

In the numerical simulation, fixed constraints were applied to the bottom face, and a 30 mm displacement load was applied through the top compression face at a loading rate of 3 mm/s. It should be noted that for the simulation model of truss-type unit cell specimens, the beam elements cannot accurately describe the structural response at rod junctions, which easily leads to simulation results significantly lower than experimental data. In this section, the local strengthening strategy from Reference [31] was adopted to address this issue, i.e., the beam elements within 7% of the length of the nodal regions in the BCC unit cell were thickened (diameter increased by 20%) to approximate the strengthening effect at beam junctions, as shown in Figure 11c.

### 4.2. Simulation Model of P-Surface Specimens

**Figure 12:**
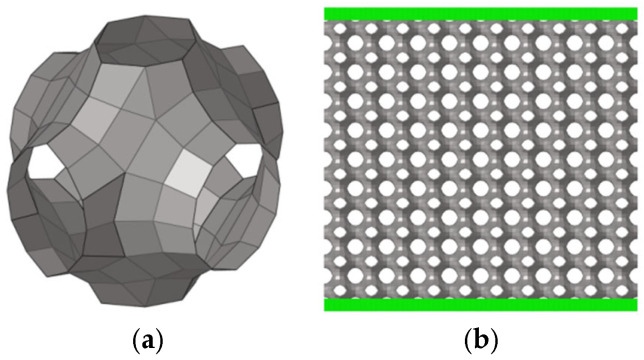
(**a**) Primitive structure unit cell; (**b**) Primitive structure lattice.

The simulation model for the P-surface lattice specimen is shown in Figure 12a. The model was established using shell elements, with a lattice region shell thickness of 0.1 mm, top and bottom face thickness of 1 mm, and a reference model with a unit cell size of 5 mm (Figure 12b). Material parameters and loading parameters were consistent with those of the BCC lattice specimen. The P-surface structure was simulated in LS-DYNA using fully integrated shell elements. The mesh density is illustrated in Figure 12.

### 4.3. Discussion

**Figure 13:**
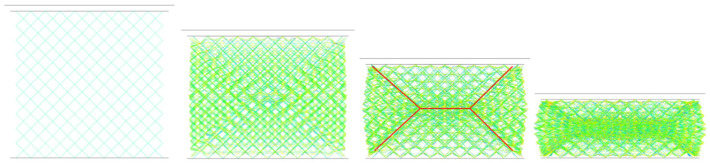
Deformation process of BCC lattice structure and axial stress distribution inside the beam.

Figure 13 illustrates the deformation processes and axial stress distributions within beams for three BCC structures, where the final deformation modes of the simulation models are generally consistent with experimental results. Simulation results indicate that the BCC lattice structure exhibits three-stage progressive deformation characteristics during compression. In the initial stage, the BCC lattice specimen (left panel) achieves uniform load-bearing through the axial force transmission of beams. As the compression displacement increases, the structure enters the plastic instability stage, where 45° shear bands first form at diagonal nodal regions, causing bending-torsion coupled deformation in beam structures and local accumulation of plastic strain. When the compression displacement continues to increase, the structure enters the densification stage, with central-region unit cells undergoing cascading collapse. Experimental observations show that the error between the measured inclination angle of the shear band (52°) and the simulated prediction (48°) is less than 8%, verifying the accuracy of the beam buckling mode. The above results demonstrate that energy dissipation in the BCC structure primarily originates from the bending-torsion deformation coupling of beam members.

**Figure 14:**
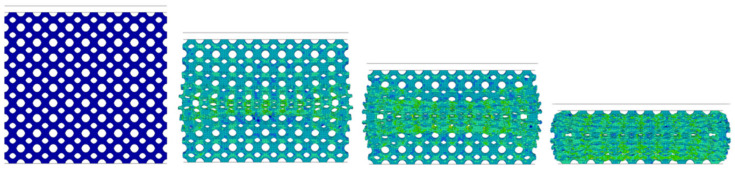
Deformation process and von Mises stress distribution of Primitive lattice structure.

Figure 14 reveals significant deformation discrepancies: the constrained structure exhibits a central-peripheral progressive deformation mode, whereas the rigid-wall-constrained structure demonstrates top-down layered yielding characteristics.

During compression, the specimen deformation generally follows a progressive mode from the center to the periphery. It can be observed that the in-plane shear force transmission mechanism of the shell structure results in a more uniform plastic strain distribution compared to the BCC structure.

## 5. Comparison of Anti-Drop Performance for Lattice-Filled Protective Structures

### 5.1. Analytical Model

**Figure 15:**
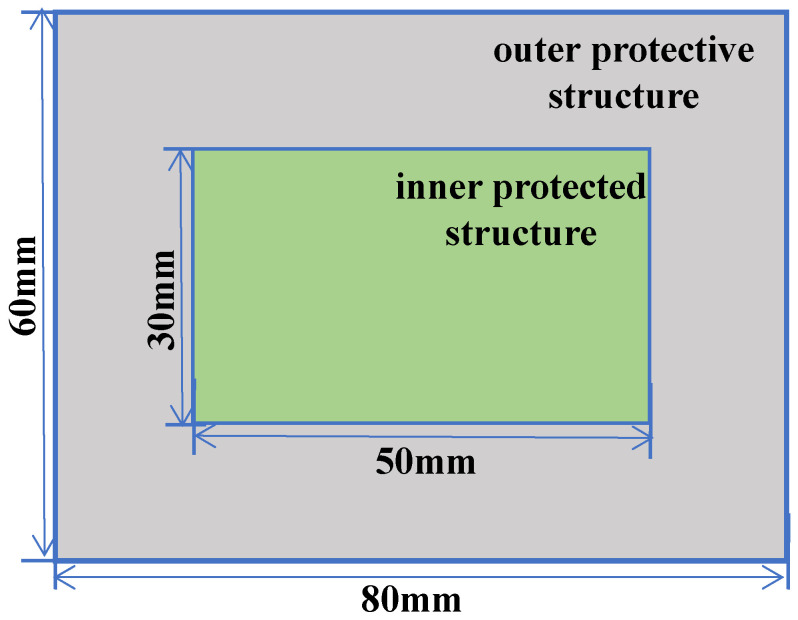
Schematic diagram of protective structure dimensions.

In this section, an impact-resistant simulation structure was designed by filling it with BCC and P-surface unit cells (as shown in Figure 15). The model consists of an outer protective structure (a rectangular cylinder of 60 mm × 60 mm × 80 mm) and an inner protected structure (a cylinder of 30 mm × 50 mm), with energy transfer paths optimized via a three-layer lattice interlayer of 5 mm unit cells. The protective skin is encapsulated by a 1 mm stainless steel shell, while the internal lattice filling layer achieves functional zoning through topological regulation: BCC unit cells with a rod radius of 0.36 mm form a bending-dominated energy dissipation zone, and P-surface unit cells with a wall thickness of 0.24 mm create an in-plane shear energy dissipation zone. The relative densities of both configurations were controlled at the same level to ensure performance comparability under mass-equivalent conditions. This design is expected to enable efficient conduction of impact energy through the skin-lattice interface, leveraging the progressive crushing of BCC and the layered collapse of P-surface for synergistic energy dissipation, thereby enhancing the impact robustness of the structure.

**Figure 16:**
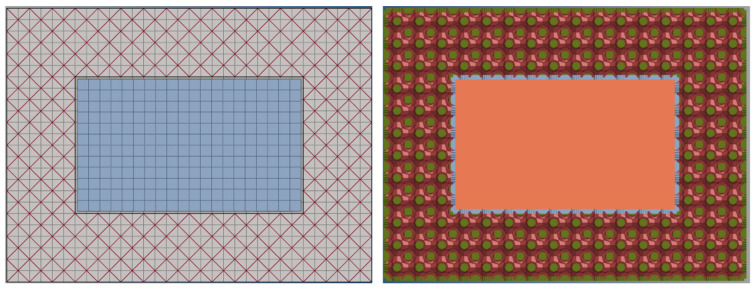
Internal schematic diagram of BCC and P-surface filling protective structure.

The numerical simulation model is shown in Figure 16, where the BCC lattice is discretized using beam elements, the P-surface configuration is modeled with shell elements, and the skin structure is accurately represented by reduced-integration shell elements with a size of 0.2 mm. A co-nodal technique is implemented at key connection points to ensure continuous stress transfer at the lattice-skin interface. The material constitutive model adopts a bilinear isotropic hardening model, with an elastic modulus of 200 GPa, yield strength of 480 MPa, and tangent modulus of 2 GPa, which fully matches the experimental material parameters (as listed in Table 1). In the drop simulation, the ground is constructed using rigid elements, the entire protective structure is assigned an initial velocity of 35 m/s, and the time step is optimized to 1 × 10^−8^ s via mass scaling technology to ensure computational accuracy while enhancing solution efficiency.

**Figure 17:**
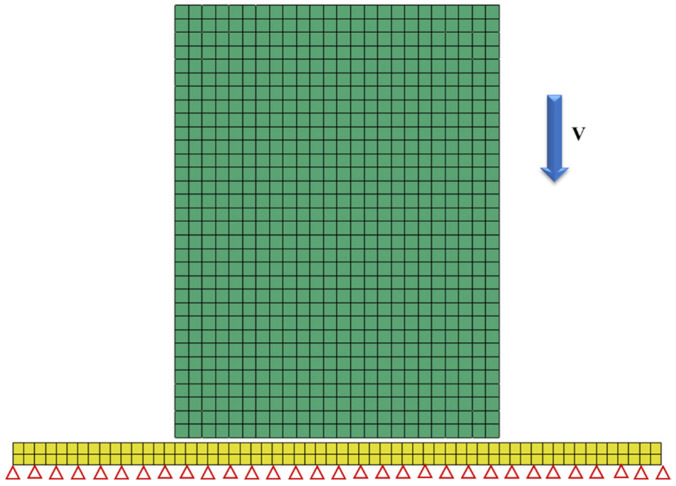
Schematic of the initial setup with a drop velocity v = 35m/s.

Fixed constraints were applied to the bottom periphery of the model to accurately replicate the actual installation boundary (Figure 17). The impact load was solved using an explicit dynamic algorithm, and the penalty function method was selected as the contact algorithm with a friction coefficient of 0.15 to characterize the actual interfacial friction effect. The data acquisition system recorded the stress–strain histories of critical regions (skin connections, unit cell cross-linking nodes) in real time, and core indicators such as the specific energy absorption (SEA) and peak impact force were quantified through an energy tracking module. The ultra-short computation duration of 0.001 s effectively captured stress wave propagation details on the order of 30 μs, providing high-precision data support for revealing the dynamic response differences between BCC and P-surface configurations.

### 5.2. Results Comparison and Analysis

**Figure 18:**
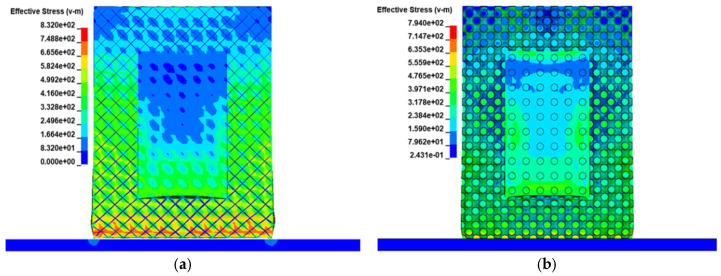
Cloud map of impact equivalent stress distribution: (**a**) BCC filled structure; (**b**) P-surface filled structure.

Figure 18 compares the Mises stress distributions of BCC-filled and P-surface-filled protective structures at the moment of maximum acceleration during impact. Results show that both structures effectively protect the internal safeguarded body, with both lattices undergoing significant deformation in the bottom impacted regions while remaining almost unaffected in other parts, validating the feasibility of lightweight design. The P-surface structure exhibits a maximum stress of 832 MPa, 4.6% higher than the BCC structure’s 794 MPa, but the area of its high-stress region (>700 MPa) is only 38% of that in the BCC structure. This phenomenon originates from the unique stress dispersion mechanism of the P-surface, where its continuous surface topology transfers impact energy to non-impacted regions via in-plane shear waves, whereas the discrete beam architecture of the BCC structure leads to local stress funnels at impact angles.

**Figure 19:**
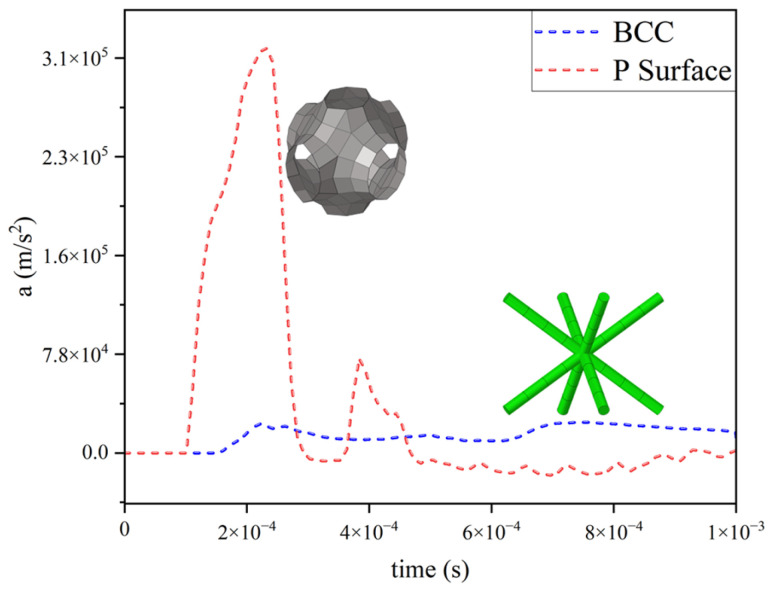
Acceleration–time history curves of the protected object for the two lattice-based protective structures.

To further evaluate the vibration-damping effectiveness of the two types of protective structures, Figure 19 presents the acceleration–time history of the protected body throughout the entire drop process. As can be observed, the P-surface structure exhibits a sharp peak during the initial impact phase, with a maximum acceleration of 3.20 × 10^5^ m/s^2^, accompanied by significant oscillation. In contrast, the BCC structure shows a gentler response, with a peak acceleration of only 2.42 × 10^4^ m/s^2^, approximately 1/13 of the former. Combined with the stress contour plots discussed earlier, it can be inferred that while the P-surface structure achieves efficient energy dissipation through in-plane shear and staged collapse, it transmits higher transient acceleration to the protected body. On the other hand, the BCC lattice demonstrates greater advantages in maintaining lower plateau forces and inducing smaller acceleration impacts.

Based on the above data comparing the comprehensive performance of the two impact-resistant protective structures: the advantages of the P-surface structure are that the enhanced stress dispersion efficiency increases the critical failure energy threshold, while the layered collapse mode enables hierarchical energy dissipation, making it more conducive to buffering control than the abrupt failure of BCC; the advantages of the BCC unit cell lie in its superior processing quality and ability to maintain low plateau force levels, making it extremely valuable in acceleration-sensitive protective structure design problems.

## 6. Conclusions

1. Manufacturing fidelity depends strongly on topology under identical SLM conditions. Although all six lattices shared the same nominal STL mass (287.6 g), the printed specimens showed clear configuration-dependent mass deviations. Truss-like lattices exhibited smaller deviations (≈0.9–10.7% across the three designs), whereas TPMS lattices showed much larger positive deviations (≈16.8–28.3%). The latter is mainly attributed to powder entrapment in semi-enclosed TPMS cavities, highlighting a practical limitation for weight-critical applications.
2. Under quasi-static compression, all lattices show the classic elastic–plateau–densification response, but with distinct plateau characteristics. Truss-like lattices generally exhibit lower plateau stress (around 50 MPa) and more stable plateau force, consistent with stress concentration at joints and the early formation of point plastic hinges. In contrast, TPMS lattices develop higher plateau stress (≈75–125 MPa) because continuous surfaces require the development of distributed line plastic hinges, often leading to plateau hardening as plastic zones expand and wall contact increases.
3. TPMS lattices provide superior mass-normalized energy absorption. The average SEA of TPMS lattices is about 2.3× that of truss-like lattices; the Gyroid achieves ~25 kJ/kg versus the best truss-like value of ~11 kJ/kg, and TPMS lattices also exhibit a longer plateau strain (≈0.52–0.57) than truss-like lattices (≈0.37–0.48).
4. Mechanistic simulations confirm topology-governed deformation modes. BCC dissipates energy via localized shear bands initiated by point plastic hinges, whereas the P-surface shows more uniform plastic deformation driven by in-plane shear and distributed hinging.

Overall, this study clarifies the design trade-off: TPMS is preferable when maximizing SEA and plateau strain is the priority and manufacturing challenges can be managed; truss-like lattices are advantageous when mass control and stable, low plateau forces are critical (e.g., acceleration-sensitive protection).

## Figures and Tables

**Table 1:** Parameters of additive manufacturing.

Material	Grade	Physical Property			
		Particle Size	Form	Mobility	Apparent Density
stainless steel	316L	15–53 μm	sphere	40 s	3.9 g/cm^3^

**Table 2:** Specimen Weight (g).

Single Cell Type	Design Weight	Weight of Experiment Item 1	Relative Error 1	Weight of Experiment Item 2	Relative Error 2
Fluorite	287.60 g	283.00 g	1.60%	284.97 g	0.91%
Diamond	287.60 g	263.14 g	8.50%	261.19 g	9.18%
BCC	287.60 g	318.34 g	10.69%	317.17 g	10.28%
G surface	287.60 g	367.08 g	27.63%	368.85 g	28.25%
P-surface	287.60 g	336.00 g	16.83%	336.00 g	16.83%
Dsurface	287.60 g	349.28 g	21.45%	347.36 g	20.78%

## Data Availability

The original contributions presented in this study are included in the article. Further inquiries can be directed to the corresponding author.
